# Comparison of fully quantitative and semi-quantitative measure of women's myocardial perfusion reserve for detection of microvascular coronary dysfunction

**DOI:** 10.1186/1532-429X-15-S1-P75

**Published:** 2013-01-30

**Authors:** David Chen, Behzad Sharif, Afsaneh Haftbaradaran, Melody Zaya, Chrisandra Shufelt, Puja K Mehta, Daniel S Berman, Louise E Thomson, Debiao Li, Noel Bairey Merz

**Affiliations:** 1 BIRI, Cedars Sinai Hospital, Los Angeles, CA, USA; 2 Northwestern University, Evanston, IL, USA

## Background

Myocardial perfusion reserve (MPR) acquired using first-pass cardiac MRI is a measure of the severity of microvascular coronary dysfunction (MCD) in subjects with signs and symptoms of ischemia but no obstructive coronary artery disease (CAD) [Shufelt 2011]. Semi-quantitative measures (upslopes) have been used to analyze MPR data in the previous work [Shufelt 2011]. In this work, we propose using a fully quantitative myocardial blood flow (MBF) analysis to evaluate whether MPR is different between subjects with MCD and normal controls.

## Methods

Control (n=9) and MCD subjects (n=10) from the Women's Ischemia Syndrome Evaluation Coronary Vascular Dysfunction (WISE CVD) multi-center National Heart, Lung, and Blood sponsored study underwent cardiac perfusion MRI studies on a Siemens 1.5T Avanto system with IRB approval and written consent. Control subjects were middle aged women with no risk factors and normal exercise ECG. Diseased subjects were middle aged women presenting with signs and symptoms of ischemia but no obstructive CAD (<50% stenosis) on angiography. A standardized first pass adenosine stress and rest first pass perfusion protocol with breath hold was performed. Segmentation (AHA) and upslope analysis were performed using CAAS MRV (PIE Medical Imaging). Only the midslice segments were analyzed to insure minimize effect of cardiac phase related MBF variations [Radjenovic 2010]. Signal intensity curves used for MBF estimation was found using the same segments and exported using CAAS MRV. MBFs were found using nonparametric deconvolution code written in MATLAB [Jerosh-Herold 2002]. A two sided Student's t-test was performed to compare groups.

## Results

Mean MPR for diseased and normal populations averaged across all AHA segments in the midslice are summarized in table 1. There is a significant difference in MPR between normal and diseased groupds using the fully quantitative MBF analysis compared to a trend using for the semi-quantitative upslope analysis.

**Table 1:** 

	Control	Abnormal, no CAD	p-value
Fully Quantitative (MBF)	2.91±0.37	2.28±0.58	0.014
Semi-Quantitative (Upslope)	2.02±0.63	1.59±0.40	0.085

Mean MPR found using MBF estimation and upslopes in control and abnormal populations. MBF parameter yielded significant difference between normal and abnormal population while upslopes did not.

## Conclusions

MPR measured by quantitative MBF analysis provides better detection of MCD than that using semi-quantitative upslopes. This finding could be explained by the observation that upslopes may not be sensitive to high blood flows [Hsu 2006], which potentially reduces the difference between diseased (ischemic) and normal tissues. In conclusion, fully quantitative MBF analysis improves detection of MCD over semi-quantitative measures. Additional work in more subjects, as well as validation with MPR measured by PET is needed.

## Funding

T32 EB51705; RO1 EB002623; AHA 11POST7390063; N01-HV-68161; N01-HV-68162; N01-HV-68163; N01-HV-68164; U0164829; U01 HL649141; U01 HL649241; T32HL69751; 5R01HL090957; 1R03AG032631.

